# Uses and Challenges of Antiviral Polyclonal and Monoclonal Antibody Therapies

**DOI:** 10.3390/pharmaceutics15051538

**Published:** 2023-05-19

**Authors:** Evi B. Struble, Jonathan M. O. Rawson, Tzanko Stantchev, Dorothy Scott, Marjorie A. Shapiro

**Affiliations:** 1 Division of Plasma Derivatives, Office of Plasma Protein Therapeutics CMC, Office of Therapeutic Products, Center for Biologics Evaluation and Research, United States Food and Drug Administration, Silver Spring, MD 20993, USA; dorothy.scott@fda.hhs.gov; 2 Division of Antivirals, Office of Infectious Diseases, Office of New Drugs, Center for Drug Evaluation and Research, United States Food and Drug Administration, Silver Spring, MD 20993, USA; jonathan.rawson@fda.hhs.gov; 3 Division of Biotechnology Review and Research 1, Office of Biotechnology Products, Office of Pharmaceutical Quality, Center for Drug Evaluation and Research, United States Food and Drug Administration, Silver Spring, MD 20993, USA; tzanko.stantchev@fda.hhs.gov

**Keywords:** antiviral therapy, antiviral antibodies, antibody combination therapy, antibody potency, potency assays

## Abstract

Viral diseases represent a major public health concerns and ever-present risks for developing into future pandemics. Antiviral antibody therapeutics, either alone or in combination with other therapies, emerged as valuable preventative and treatment options, including during global emergencies. Here we will discuss polyclonal and monoclonal antiviral antibody therapies, focusing on the unique biochemical and physiological properties that make them well-suited as therapeutic agents. We will describe the methods of antibody characterization and potency assessment throughout development, highlighting similarities and differences between polyclonal and monoclonal products as appropriate. In addition, we will consider the benefits and challenges of antiviral antibodies when used in combination with other antibodies or other types of antiviral therapeutics. Lastly, we will discuss novel approaches to the characterization and development of antiviral antibodies and identify areas that would benefit from additional research.

## 1. Introduction

Infectious diseases are a major global health burden with eight major diseases—HIV/AIDS, malaria, measles, hepatitis, dengue fever, rabies, tuberculosis and yellow fever—exacting a heavy toll in terms of human lives lost [1]. The coronavirus disease 2019 (COVID-19) pandemic further exacerbated the cost to human life and long-term health outcomes. Emerging and re-emerging viral diseases, such as Ebola, Zika, Lassa fever, measles, highly pathogenic avian influenza, etc., continue to pose a risk not only for local/regional outbreaks, but also for becoming the next pandemic. The availability of safe and effective prophylaxis and treatment options for these and other infectious diseases is a top public health priority. Antibody therapeutics have long been used in viral disease settings; for example, post-exposure prophylaxis for rabies or hepatitis B with respective hyper- or specific-immune globulin (IG, also known as immunoglobulin), or the use of monoclonal antibody (mAb) therapies for the prevention of respiratory syncytial virus (RSV) infection. Recent approvals of mAb therapies for human immunodeficiency virus type-1 (HIV-1) and Ebola virus (EBOV), as well as the rapid development and emergency use authorization of several mAbs against severe acute respiratory syndrome coronavirus 2 (SARS-CoV-2) for prophylaxis and treatment of COVID-19, further highlight the potential of these molecules, either alone or in combination with other therapies, to make a significant impact on public health. In this review, we will discuss the biochemical and physiological characteristics that render antibody molecules desirable therapeutics, pre-clinical assays that can be used to assess potency, discuss the benefits and challenges of antibody combination therapies, and highlight areas in need of additional research.

## 2. Antibodies as Therapeutics

With very few exceptions, antibody therapeutics approved to date are isotype G immunoglobulins (IgG). IgGs are protein macromolecules secreted in the blood of most vertebrates [2] through differentiated plasma B cells that have a high affinity and specificity for their respective antigen. The IgG molecules can then be purified from human or animal plasma to produce polyclonal immune globulin products. These types of products, such as diphtheria antitoxin [3], represent some of the first products to be licensed in the United States. In over a century of development, polyclonal products underwent tremendous advances in the manufacturing process and characterization of safety and efficacy attributes. In the last few decades, antibody therapeutic development shifted toward the development of IgG monoclonal antibodies that are engineered for in vitro expression in mammalian cell lines. Candidate antibodies are identified via traditional hybridoma technology, as well as increasingly through using mice engineered to express human VH and VL genes [4], phage or yeast display technologies [5], isolating virus antigen specific B cells from convalescent patients [6,7,8,9], or a combination of these technologies [10].

**Figure 1:**
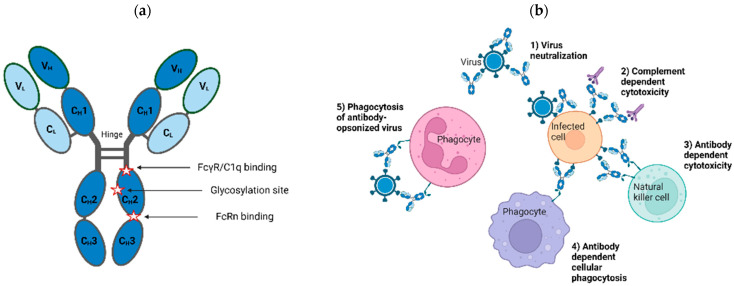
Structural and functional features of isotype G immunoglobulins (IgG). (**a**) Structural features of IgG antibodies. IgG macromolecule is a tetramer of two identical heavy (H) and light (L) chains depicted in dark and light blue, respectively, each containing variable (V_H_, V_L_) and constant (C_H_, C_L_) regions, as shown. Glycosylation site and locations responsible for receptor and complement binding are marked. These regions can be engineered to modulate downstream properties of IgG products. (**b**) Antiviral functions of IgG antibodies. Antiviral pharmacologic properties of antibody therapies are as follows: (1) neutralization of viral entry to its cell target; (2) complement- and (3) antibody-mediated cytotoxicity of infected cells; (4) phagocytosis of infected cells; and (5) clearing of opsonized virus through phagocytosis. Figure created with BioRender.com, accessed on 17 May 2023.

The structural and functional features of IgG antibodies render them well suited for use as therapeutics. Structurally, the molecule can be thought of as modular, with two identical heavy chains (HC) and two identical light chains (LC). The IgG HC comprises four domains: one variable (V) domain and three constant (C_H_1, C_H_2, and C_H_3) domains, with a hinge region between the C_H_1 and C_H_2 domains (Figure 1a). The LC comprises two domains: a variable (V) domain and a constant (C_L_) domain. The fragment antigen binding (Fab) region in each chain contains both V and constant (C_H_1 or C_L_) domains, with the former housing the complementarity determining regions (CDR) responsible for epitope recognition and antibody specificity. When properly folded, the CDRs of the HC and LC come together to form the antigen-binding site. The fragment crystallizable (Fc) region, comprising the HC C_H_2 and C_H_3 domains, is responsible for downstream processes (Fc effector functions) that result in immune activation and the ultimate destruction of the antigen. There are four different IgG subclasses (IgG1, IgG2, IgG3 and IgG4), with respective polymorphic variants [11]. Each subclass has different affinities for Fc receptors, which impacts their ability to engage different effector cells and mediate effector functions [12]. Most mAbs, including those directed against viral diseases, belong to the IgG1 subclass, since IgG1 antibodies have long half-lives and can efficiently mediate a wide variety of effector functions. In addition, IgG antibodies have a single N-glycan in the constant region. These biochemical properties (i.e., sequence and glycan structures) play an important role in physicochemical (i.e., stability, shelf-life), pharmacokinetic, and pharmacodynamic properties of the antibody therapeutic, and, thus, should be well characterized during development.

The use of IgG products as prophylactic and therapeutic modalities for viral diseases is predicated on their ability to bind to one or more antigens on the surface of viral particles and/or infected cells via the antigen-binding sites. They can neutralize the ability of viruses to enter cells through blocking attachment or fusion, inactivating/disrupting virus particles, or triggering the killing of infected cells through Fc-mediated effector functions (Figure 1b; see [13,14,15,16] for a comprehensive review of the mechanism of virus neutralization). For the latter function, the antigen-antibody complex is recognized by effector molecules, such as the C1q component of complement or Fc gamma receptors (FcγRs) present on the surface of effector cells, giving rise to immune signaling cascades that culminate with the clearance of viruses and/or infected cells. In some cases, Fc effector functions are shown to enhance the antiviral activity of specific antibodies [16,17,18,19,20].

On the other hand, antibody-dependent enhancement (ADE) of viral infection or disease can also occur [21], as has been well documented in humans for dengue virus [22]. ADE can arise after natural infection, vaccination, or passive transfer of antibody therapies. It is widely thought that ADE occurs when antibodies of insufficient avidity or concentration are unable to neutralize the virus, but can facilitate the uptake of the virus-antibody complex through FcγR-bearing cells, such as monocytes, dendritic cells, or macrophages [23], resulting in increased viral production, enhanced immune activation (e.g., cytokine production), and more severe disease cases [24]. In addition to flaviviruses [25,26], ADE is observed for mAbs against influenza virus, HIV-1, and EBOV in cell culture, but not typically when tested in animal models or clinical trials, with a few exceptions [23,25]. When selecting antibodies best suited for use as an antiviral product, it is critical to optimize binding both to the antigen and FcγRs. For mAbs, the risk of ADE can be reduced through selection of a particular IgG subclass [27], modification of Fc glycans, or engineering substitutions into the Fc region that disrupt FcγR binding; however, these substitutions may also disrupt Fc effector functions that could contribute to clinical efficacy [28,29]. Although ADE in cell culture and animal studies was observed with antiviral specific polyclonal IGs [30], clinical ADE was not reported to our knowledge for any FDA-approved specific IG products.

During pharmaceutical development, mAb domains often undergo extensive biochemical engineering to optimize the properties of the antibody. For example, to humanize mAbs derived from mice or other species, the CDRs can be grafted onto the framework regions of V domains from other mAbs or germline V genes while retaining their antigen-binding properties in the context of a known protein fold [31]. In general, all mAb V regions are engineered to improve manufacturability and stability and optimize binding [32]. The Fc region can also be modified to alter pharmacokinetic properties and effector functions. On the other hand, although not subjected to Fc engineering, depending on the antigen or donor population, specific antiviral polyclonal IGs can be “enriched” for a particular isotype [33], subclass, or glycosylation signature, leading to different Fc effector functions compared to other polyclonal IG products. For example, IgG1 and IgG4 are the most prevalent subclasses following measles infection or vaccination, with significant differences in titers in infected versus vaccinated individuals [34]. In addition, anti-SARS-CoV-2 antibodies from convalescent donors have distinct glycosylation patterns depending on disease severity [35]. We will discuss some of the methods currently used to design, produce, and characterize antibody products, highlighting the differences between polyclonal and monoclonal antibody therapies.

### 2.1. Production and Characterization of Antibody Therapies

#### 2.1.1. Specific Polyclonal Antibody Therapies

Specific polyclonal IG (SpIG) is used as the overarching term for all polyclonal preparations that are enriched for certain antiviral, antibacterial, or antitoxin antibodies. SpIGs are purified from plasma of humans who were vaccinated or recovered from a specific infection or animals that were vaccinated with a specific pathogen preparation or toxin. The first products were developed in 1898 and comprised little more than serum from horses vaccinated with virus preparations, bacterial toxins, or snake venom. In 1903, diphtheria antitoxin made from vaccinated horses became the first licensed product in the United States.

SpIGs from animal sources are produced via repeatedly immunizing donor animals. Advantages of large animal donors (horses, sheep, or cattle) include the ability to immunize more frequently (which increases the yield and avidity of specific antibodies), use experimental vaccinations, and safely collect larger volumes of plasma. A major disadvantage includes potential allergic reactions in patients due to animal proteins, including the antibodies. Animal-derived antibodies are often treated with pepsin or trypsin to remove the Fc portion and reduce immunogenicity. These fragments lack effector functions that could be important for antibody activity, depending upon the virus. An interesting strategy has been developed using transchromosomic cattle that produce full-length human IgG antibodies. The cattle are knocked out for bovine antibody heavy and lambda light chains, but contain an artificial chromosome encoding the respective human IgG chains. Chimeric antibodies consisting of human IgG heavy chains and bovine kappa light chains are removed during manufacturing [36]; thus, the resulting IG product manufactured from these bovines contain only human IgGs, lowering the risk of immunogenicity. These transchromosomic bovines were successfully hyperimmunized [37].

Research during World War II stimulated a major breakthrough in purification of IGs and other proteins from human plasma. IG purification methods are usually based on sequential alcohol precipitations, each with specific conditions of pH, ionic strength, temperature, protein concentration, and alcohol concentration [38,39]. For some products, purely chromatographic methods or caprylate precipitation methods have partially or completely supplanted alcohol precipitation. These changes are often driven by the need to increase yield of IgG, thus increasing product availability [40]. Nevertheless, alcohol-based fractionation remains the backbone of early steps in production of most IG products and is often combined with subsequent caprylate or polyethylene glycol precipitations. Modern IG products are further purified using column chromatography to remove unwanted plasma proteins or viral inactivating compounds used for upstream manufacturing steps. A minimum of two orthogonal, robust, dedicated viral clearance steps are performed, which often include solvent-detergent treatment and nanofiltration, as well as other virucidal (caprylate, heat treatment, low pH) and partitioning (chromatography, precipitations, depth filtration) steps. All viral clearance steps must be validated and found to be robust using scaled-down models of the manufacturing process and actual manufacturing intermediates spiked with virus as starting material. It should be emphasized that modern IG purification is highly complex with multiple steps, each of which must be controlled to result in a safe and intact product. Every manufacturing method is unique with respect to purification details and methodology (such as mixing speeds, equipment used, precipitation times, buffer types and concentrations, centrifugation vs. precipitation), as well as the equipment. Thus, each product is also unique with respect to levels and types of plasma protein impurities and IG stability.

Antibody enrichment for human antibodies is achieved through either immunizing donors or screening and selecting high-titer plasma from routine donations (as for Cytogam [41]) or convalescent donors (as for early versions of SARS-CoV-2 IG investigational products [42,43]). “Hyperimmune” polyclonal antibodies are derived from donors who were immunized intentionally for the purpose of obtaining high-titer plasma (e.g., rabies, vaccinia, or hepatitis B in humans). Nevertheless, convalescent plasma is often inaccurately referred to as “hyperimmune,” even though donors were not immunized. Under FDA-approved plasma center collection protocols, and after investigational safety studies are completed, hyperimmune plasma can be collected from consenting immunized donors. Human-derived, antiviral SpIG products licensed in the United States are shown in Table 1.

For purposes of final product testing, a validated bioassay demonstrating neutralization in cell culture or in animals is ideally performed for SpIG products. In special cases, adequate cell culture or animal models are not available at the time of licensure. In this situation, a binding assay is usually selected and validated for product release, contingent on discussions with FDA. Likewise, national or international IgG standards may be lacking. In these instances, an internal IgG standard is developed by the manufacturer.

##### Treatment Timing and Dosing for SpIG

Treatment timing relative to infection depends on demonstrable efficacy of the product for pre- or post-exposure prophylaxis. Pre- and post-exposure prophylaxis can be effective (if adequately dosed) largely because viral burdens are relatively low during early infection. Even if an infection was initiated, post-exposure prophylaxis attenuates disease severity of measles, HAV, and varicella zoster [47,53]. When vaccines are given concomitantly with specific IG, such as for rabies, passive immunization provides a defensive “bridge” that acts immediately to neutralize the virus until vaccine responses arise. It is important that the dose of rabies IG (RIG) is not so high that it suppresses the vaccine response. In such contexts, both a minimum and maximum potency should be defined to assure optimal function of both RIG and the vaccine. Pharmacokinetic studies performed in healthy immunocompetent human subjects are used to define the dose of SpIG that is needed to avoid suppression of vaccine responses, while still being able to provide protection until vaccine responses are sufficiently developed.

Treatment of symptomatic viral disease with SpIG is much more challenging and often ineffective. In these cases, the viral burden may exceed the capacity of the IG, viruses may be relatively inaccessible within infected cells or immune-privileged sites, and cellular immune responses may also be suppressed by the virus [59]. Notable lack of efficacy via specific IG for treatment of symptomatic infections, such as rabies, influenza, HAV, HBV, measles, and varicella, were observed. The time windows for effective post-exposure prophylaxis of each infection were established based on such failures. Treatment with CMVIG and HBVIG(IV) can prevent severe disease in transplanted patients but are not curative. Vaccinia Immune Globulin is used to treat severe complications (eczema vaccinatum and progressive vaccinia) resulting from live vaccinia virus vaccine (ACAM2000), which is used to prevent smallpox. The recently licensed replication-deficient vaccinia virus (Jynneos) also generates an immune response, and is thought to be incapable of causing eczema vaccinatum or progressive vaccinia. Both vaccines are indicated for prevention of smallpox. Jynneos is also licensed for prevention of monkeypox [60].

#### 2.1.2. Monoclonal Antibodies

To date, the FDA has approved four mAb therapies to prevent or treat viral diseases (Table 2): palivizumab for prevention of RSV in pre-term infants and infants with other specific conditions, ibalizumab for treatment of HIV-1 in patients failing their current anti-retroviral regimen, and two products for treatment of Ebola virus disease resulting from *Zaire ebolavirus*. One of these products, known as Inmazeb, consists of three mAbs that target non-overlapping epitopes on EBOV glycoprotein, and represents the first co-formulated mAb cocktail approved by the FDA [61].

Multiple mAbs are currently either in advanced stages of clinical development or were approved in other countries. Nirsevimab, which is a half-life extended mAb that targets the RSV fusion (F) protein [62], was recently approved by the European Medicines Agency for the prevention of RSV lower respiratory tract disease in neonates and infants during their first RSV season. In addition, three mAb products targeting the rabies virus glycoprotein were approved in other countries: two in India (Rabishield, a single mAb, and TwinRab, a cocktail of two mAbs [63]) and one in China (ormutivimab [64]).

Several mAbs and mAb combinations that target the SARS-CoV-2 spike protein were rapidly developed after the onset of the COVID-19 pandemic, and received emergency use authorization (EUA) from the FDA for the pre-exposure prophylaxis, post-exposure prophylaxis, and/or treatment of COVID-19. Although highly effective against early SARS-CoV-2 variants, these products are not currently authorized in the United States due to the emergence and widespread circulation of variants that are resistant to neutralization through these mAbs in cell culture [65,66,67,68,69,70,71]. However, if future variants emerge that are susceptible to these products, their authorization status may change. Refer to the FDA website for updated information on the status of EUAs for mAbs and other COVID-19 therapeutics [72].

In addition to the approved and previously authorized mAbs and those directed against SARS-CoV-2, many other mAbs were or are under development that target existing and emerging diseases [8,73,74,75].

Historically, therapeutic mAbs were derived from immunized mice or rats and engineered as chimeric (V regions from the original mAb expressed with a human constant regions) or humanized (CDRs from the original mAb grafted on to a human V region backbone) mAbs to reduce the immunogenicity due the “foreignness” of rodent mAbs in humans. Currently, most mAbs are of human origin, being derived from either “humanized” mice or other species that express human germline V(D)J region genes or from phage display libraries generated from human donor lymphocytes. However, many antiviral mAbs are isolated directly from previously infected patients [6,7,8,9]. Regardless of the source, many considerations inform the selection and engineering of candidate mAbs.

##### Engineering of mAbs

Most mAbs developed for viral diseases are, firstly, selected for their ability to neutralize virus entry. However, Fc effector functions play a major role in the immune system’s response to infectious diseases [19]. For mAbs, the contribution of Fc effector functions to disease protection were demonstrated in non-clinical studies for several viruses, including Ebola virus [16], HIV-1 [17,76,77], influenza [78], SARS-COV-2 [79], and Rift Valley fever virus [80]. However, ADE of infection or disease is a possible negative consequence of FcγR binding [21,81]. Therefore, depending on what is known about specific viral diseases, different approaches can be used to engineer the Fc region of mAbs to either enhance or diminish FcγR binding. Amino acid residues were identified in the IgG Fc region that contact the complement component C1q; FcγRs; or the neonatal Fc receptor (FcRn), which is responsible for the long half-life of IgG [82,83]. Substitutions can be engineered at these residues to alter Fc effector functions or extend the half-life of a mAb, which allows less frequent dosing [84]. The half-life of mAbs can also be extended through engineering the Fab region to alter its pH-dependent antigen binding properties, which, upon internalization, leads to antigen dissociation in acidic endosomes and subsequent degradation in lysosomes, while the unbound mAb is recycled back into circulation [85,86]. When combined with Fc modifications to extend antibody half-life, this type of engineering approach can greatly reduce antigen concentrations in plasma [87].

In addition to Fc engineering, there is a better understanding of specific Fc glycan structures and their association with different effector functions, e.g., afucosylated mAbs, which have better antibody dependent cellular cytotoxicity (ADCC) compared to highly fucosylated antibodies, while galactosylation is associated with complement dependent cytotoxicity (CDC) and can influence ADCC activity [88]. Furthermore, mAbs produced in cell culture have considerable heterogeneity in glycosylation patterns. Therefore, cell lines were engineered to produce mAbs with up to 100% afucosylation to enhance ADCC activity [82,89]. The understanding of the relationship between antibody glycan structures and Fc effector functions is ongoing, and additional strategies may be developed to further engineer mAb glycan structures. For example, the effect of galactosylation on ADCC activity may depend on the specific linkage of the galactose monosaccharide [90]. Fc effector functions can be reduced through introducing substitutions at the glycosylation site (N297) in the CH2 domain to prevent the addition of a glycan [91,92], thus providing another glycoengineering approach for antiviral mAbs.

##### Development of mAb Combinations

Three of the four approved monoclonal antiviral products are single mAbs; however, the anti-Ebola virus mAb cocktail of atoltivimab, maftivimab, odesivimab-ebgn was the first fixed dose co-formulated mAb combination product approved by the FDA. Many other mAbs are used in combination to treat viral diseases and for other indications, but only a few to date are co-formulated [93]. The advantage of antibody cocktails over a single mAb is that they might be less susceptible to escape, depending on the different targeted epitopes. As seen for the anti-SARS-CoV-2 mAb combinations previously authorized for the prophylaxis or treatment of COVID-19, they all target the SARS-CoV-2 receptor binding domain of the SARS-CoV-2 spike protein, but have little neutralization activity against current variants. MAbs that target regions outside the receptor binding domain could neutralize virus or mediate Fc effector functions and might be less susceptible to escape. For example, a recent report demonstrated that mAbs targeting the conserved fusion peptide region adjacent to the S2′ cleavage site of the spike protein are broadly neutralizing against betacoronaviruses [94].

### 2.2. Advantages and Disadvantages of Polyclonal and Monoclonal Antibodies

There are advantages and disadvantages when selecting a product for treatment or prophylaxis of viral diseases, some of which are summarized in Table 3. For approved products, the choice is often based on which products are available for a specific viral disease. For example, currently only SpIG products are approved in the United States to treat rabies, CMV, HBV, varicella or vaccinia, whereas only mAb therapies are approved to prevent or treat RSV, HIV-1, and EBOV disease. There are other considerations that also play a role in the development or deployment of antibody therapies in an infectious disease setting. Although resistance to polyclonal antibodies is reported [95], polyclonal antiviral products are less likely to result in treatment-emergent resistance or the formation of an antibody response to the treatment (anti-drug antibodies), whereas both issues are a larger concern for mAb products. On the other hand, given the relative ease of engineering, development, and production of mAbs, they are well suited for rapid development, especially in an emerging infectious disease setting. Both types of products can have drawbacks that include the potential to interfere with the immune response to the vaccine or natural infection, as well as specific diagnostics, and the potential to result in enhanced infection or disease, as already described. Despite these limitations, the benefit-to-risk ratio for these approved products is favorable, as demonstrated in clinical trials and through routine clinical use in viral disease settings.

## 3. Evaluation of Antiviral Activity

Prior to being evaluated in clinical studies as antiviral therapies, biological activity and potential mechanisms of action for antibodies are investigated in pre-clinical studies performed in model systems. In practice, assays to assess antibody activity usually fall into three broad categories: biochemical (e.g., binding) assays, cell culture assays, and animal models. Early in the pharmaceutical development of the antibody therapies, these assays are performed as part of candidate selection and then to characterize the antibody product that is being developed. Multiple such assays can be performed with the goal of understanding different aspects of antibody antiviral activity. Examples include antibody binding affinity, epitope characterization, neutralization activity, and assays to characterize Fc effector functions. Some of these assays will be developed as quality control potency assays to ensure the lot-to-lot consistency and stability of the product. Federal regulations define potency as “the specific ability or capacity of the product, as indicated by appropriate laboratory tests or by adequately controlled clinical data… to effect a given result.” (21 CFR. 600.3(s)). Thus, for antiviral antibody therapies, potency assays provide a quantitative measure of the antibody activity linked to its primary mechanism of action. Fit-for-purpose potency assays are often performed prior to initiation of Phase 1 clinical studies, and full validation is completed by the time of a biologics license application (BLA) submission. FDA guidance describing current thinking on the development and validation of such studies for mAbs was available in draft form at the time of writing this article [96]. Some points to consider when designing pre-clinical studies to evaluate antiviral activity and assess potency are discussed below.

### 3.1. Types of Potency Assays

SpIGs and mAbs may exert their antiviral effects via one or more potential mechanisms: virus neutralization, ADCC, opsonization and phagocytosis, complement lysis, and/or complement dependent cytotoxicity [13,14,15]. There are just a few examples of antibodies potentially acting at virus post-entry steps [14,15], but their role in the overall antiviral humoral immune response is yet to be established. If SpIGs or mAbs under clinical development have multiple mechanisms of action, multiple assays are developed and implemented for quality control. In general, the selected potency methods should reflect the product’s proposed mechanism as closely as possible. Potency is usually evaluated via a comparison to an appropriately qualified reference standard and expressed as a percentage of the reference material value. For SpIGs, international or national standards are often used, e.g., for anti-rabies, anti-hepatitis B, or anti-measles IGs. The potency is then expressed in international or alternative units, as appropriate. As for all quality control release methods, key assays for demonstrating the antiviral mechanism(s) of action should be shown to be suitable for their intended purposes during development and validated by the time of an application for approval. Ideally, potency assays which adequately reflect the proposed mechanism(s) of action should be qualified and implemented before pivotal clinical trials [96].

Antibody–antigen binding is a necessary step for both virus neutralization and Fc effector functions. Therefore, binding assays, such as an enzyme-linked immunosorbent assay (ELISA) or a surface plasmon resonance (SPR) assay, are a logical approach to evaluate drug potency. There is significant experience with these types of assays, and they may be easier to qualify and validate compared to cell-based methods used to assess the antiviral activity of therapeutic antibodies. In general, potency-binding assays are developed and used during the early stages of product development. However, direct binding assays may not provide a comprehensive assessment of the product’s mechanism of action. For antibodies targeting virus-cellular receptor(s) interactions, inhibitory binding assays (ELISA or SPR) may better reflect their mechanism of action, but even these assays may not fully represent the antibody-mediated suppression of the complex virus-cell fusion process. Furthermore, broadly neutralizing antibodies may target complex, conformation-dependent, and non-linear epitopes, which can be challenging to reproduce in a binding assay.

In comparison to binding assays, cell-based methods can provide a more comprehensive assessment of antibody-mediated antiviral activity, either via virus neutralization and/or Fc effector functions [96].

There is already significant expertise with the development and validation of cell-based ADCC potency assays for a variety of mAbs for the treatment of different neoplasms [97], while the qualification/validation of methods to evaluate the ADCC activity of antiviral antibodies follow the same general principles. However, challenges remain regarding the selection and qualification of relevant target and effector cells employed in these assays, which are discussed later in the manuscript.

Virus neutralization assays can employ authentic (wt) viruses, replication-competent pseudotyped virions, or cell-fusion capable but replication-incompetent pseudotyped virus-like particles (VLPs). Pseudotyped viruses and VLPs are considered safer alternative methods for studying a growing number of viruses which pose enormous health and socioeconomic risks because of their high pathogenicity, including Ebola, Sudan, Marburg, Hendra and Nipah viruses, as well as SARS and MERS, or their capacity to cause a widespread pandemic, such HIV-1, SARS-CoV-2, and certain influenza virus A subtypes. Furthermore, highly pathogenic viruses require biosafety level-3 (BSL-3) or BSL-4 facilities, which have high costs and limited availability, impeding the successful development of new therapeutic modalities.

Pseudotyped viruses, also referred to as chimeric viruses [98], are typically generated through replacing the gene(s) expressing the surface glycoprotein(s) of a virus with low pathogenicity (e.g., vesicular stomatitis virus) with the gene(s) encoding the envelope (Env) glycoprotein(s) of a BSL-3 or a BSL-4 pathogen (e.g., EBOV), thus creating a replication-competent virus that can be used in a BSL-2 environment [98,99,100,101,102,103]. However, in the case of HIV-1, replication competent, infectious pseudotyped viruses were created within the same species through replacing the original envelope gene with the one from a different HIV-1 strain, usually for the purpose of studying virus tropism and/or neutralization susceptibility [104,105,106,107,108,109]. In general, in vitro infection with either wt or pseudotyped replication competent viruses involves a self-spreading infection among the target cells, unless the time of the assay is shortened by design. Reporter genes are often inserted into the genome of pseudotyped viruses to assess the level of infection [98,102,103,109,110,111] as an alternative to measuring viral proteins, nucleic acids, or cytopathic effects [99,106,108,112]. Target cells, which are stably transfected to express the reporter gene(s) when infected, can also be used to quantify virus infection and assess the activity of antivirals, including neutralizing antibodies [109,113,114,115,116,117].

In addition to pseudotyped viruses, fusion-competent but replication-incompetent VLPs can be used to assess virus fusion and entry. VLPs are produced via co-transfecting producer cells (usually 293T cells) with the plasmid(s) encoding the desired virus surface glycoprotein(s) and the plasmid(s) encoding viral proteins necessary for VLP production. In general, VLP contain either an incomplete or no viral genome, which render them capable of just a single round of virus entry, followed by partial or no virus replication. Currently, VLPs are successfully generated for both enveloped and non-enveloped viruses [110,118,119,120,121,122,123,124,125,126,127,128]. For the generation of enveloped VLP, retroviral- (HIV-1 or murine leukemia virus derived) or rhabdoviral (VSV)-based packaging vector systems are commonly used [118,127], although other vectors are also described [109,116,129]. As with the pseudotyped viruses, to facilitate the assessment of VLP cell fusion and entry, VLPs are often engineered to include a reporter gene encoding an enzyme or a fluorescent protein (luciferase, alkaline phosphatase, β-galactosidase, green fluorescent protein), where expression reflects the level of infection [105,107,110,111,121,122,130].

Alternatively, VLPs can be used to infect stably transfected cell lines containing a reporter gene under the control of a viral regulatory protein [131,132,133]. The TZM-bl cell line, stably transfected with the luciferase and β-galactosidase genes under the control of the HIV-1 long terminal repeat promoter, which is activated using the HIV-1 tat protein, is probably the best-known example of this approach [103,114,117,132,134].

A more elaborate VLP system, based on EBOV minigenomes that encode a reporter gene, was designed to study almost all aspects of the EBOV life cycle in a BSL-2 environment [120,135]. This system may potentially be used for the development and/or screening of anti-EBOV antibodies, but its applicability for this purpose remains to be demonstrated.

It should be noted that for reporter gene encoding virus particles (wt/pseudotyped viruses or VLPs), the reporter gene expression depends not only on the virion-cell fusion, but also on post-entry events leading to the synthesis of the encoded protein. To solely study the viral cell entry process, replication competent virions or VLPs were designed to incorporate an enzyme or a fluorescent protein that is expressed in producer cells. This result is achieved through utilizing vectors that encode chimeric molecules, consisting of the “reporter protein” fused to a viral protein, which directs the entire molecule into the budding virions [120,136,137,138,139,140,141]. The assays that employ the “reporter protein” containing virions can be valuable tools to study virus entry inhibitors, but currently there are limited data, compared to the reporter gene-based methods, regarding their use for the assessment of virus-neutralizing antibodies [140].

A broad range of enveloped viruses belonging to different families and including human pathogens can induce cell–cell fusion between infected cells and neighboring non-infected cells [142]. This phenomenon serves as the basis for the development of assays measuring the level of fusion between virus surface glycoprotein-expressing cells (effector cells) and cells expressing the relevant virus receptor(s) (target cells) via quantitation of giant, multinuclear cells (syncytia) formation, fluorescent dye transfer, or reporter gene expression. The virus envelope-mediated cell–cell fusion assays are a useful tool for assessing virus–cell fusion inhibitors, including neutralizing antibodies, as a rapid surrogate for the virus entry methods. Moreover, akin to the pseudotyped and VLP systems, cell–cell fusion assays allow studies of BSL-2 environment of viruses that are otherwise restricted to a higher level of biocontainment (BSL-3 or BSL-4) [115,143,144,145,146].

The variety of methods which can be used to evaluate the effects of neutralizing antibodies raise the issue of how these methods compare to each other regarding their sensitivity and ability to predict a correlation between in vitro and in vivo results. To address this issue, efforts were made to apply a standardized approach among different labs for the assessment of antibody-mediated virus neutralization [132,147,148].

In general, multiple in vitro binding and cell-based methods are used for antibody characterization during product development, with the goal of defining the antibody’s mechanism(s) of action and the critical quality attributes potentially affecting its anti-viral activities, as both the neutralization and Fc-mediated effects of the antibody are being investigated. Assays employing wt infectious viruses are likely to remain an important part of product characterization and serve as a basis for comparison with the alternative virus neutralization methods. However, as mentioned earlier, biosafety concerns may limit the use of authentic BSL-3 and BSL-4 pathogens. The use of pseudotyped viruses and/or VLP can offer less restrictive biosafety requirements and may facilitate antibody characterization in several additional ways: a. pseudotyped viruses/VLP can be more readily manipulated, allowing faster assessment of potential substitutions in the virus surface glycoproteins; b. the level of entry may be easier to quantify; and c. panels of pseudotyped VLP, representing a wide range of virus strains that are generated using the same packaging system, can be created and tested for their neutralization susceptibility [134,149,150,151]. Ideally, the pseudotyped viruses or VLP should closely resemble the corresponding authentic viruses, but certain differences may exist regarding shape, glycosylation, and density of the envelope glycoproteins due to the packaging system and/or producer cells used. The incubation times, readout methods, and target cells may also be different [148,152]. However, similar neutralization sensitivity was demonstrated when replication competent HIV-1 and HIV-1 Env pseudotyped VLP (generated using an HIV-1 derived packaging system) were both produced in 293T cell line and tested on the same target cells [107,114]. Moreover, similar neutralization profiles were observed for spike protein pseudotyped VLP and authentic SARS-CoV-2 in Vero E6 target cells [98].

Usually, one or more of the characterization methods are adapted to become the potency assay(s) for the purpose of drug substance and drug product release and stability testing. Development and implementation of the adequate potency assay(s) is a critical quality control measure to ensure that each lot is consistently produced with the potency necessary to achieve clinical efficacy, and that such potency is maintained over the shelf life of the product. Data regarding the validation of certain commonly used pseudotyped virus/VLP assays were previously published [109,117]. It should be emphasized that adequate qualification of the assays’ critical reagents is an integral part of the validation process. Detailed information regarding the generation, quantitation, and stability of the virus/VLP stocks should be provided. Determination of the ratio of functional (infectious or fusion-capable) to non-functional virus/virus-like particles may also be important for qualification of the virus stocks [148]. Finally, control measures should be in place to ensure consistency in the performance of the target cells in the virus neutralization assays or both the target and effector cells in the virus envelope-mediated cell–cell fusion assays [96].

### 3.2. Cells for Potency Assays

For most viruses, there are a wide variety of cell lines that can be used to assess the potency of polyclonal and monoclonal antibodies with antiviral activity. For example, for SARS-CoV-2 alone, cell lines that have been used to assess antibody neutralization activity include Vero/Vero E6 (African green monkey kidney), 293/293T (human embryonic kidney), HeLa (human cervical carcinoma), Huh7 (human hepatocellular carcinoma), Calu-3 (human lung carcinoma), HT1080 (human fibrosarcoma), U2OS (human osteosarcoma), and HOS (human osteosarcoma) cells, which are often engineered to stably express human angiotensin-converting enzyme 2 (ACE2), which is the SARS-CoV-2 receptor, and/or transmembrane serine protease 2 (TMPRSS2), which is a protease involved in SARS-CoV-2 entry [65,67,70,153,154,155,156,157,158].

When selecting an appropriate cell line for the evaluation of antibodies, a large number of factors can be considered: physiologic relevance, activity to be measured (e.g., neutralization, ADCC, etc.), desired assay throughput and readout (e.g., plaque formation), feasibility of using primary cells in lieu of immortalized cell lines, expression of host factors, tissue and species origins, and potential for amino acid polymorphisms in the receptor or coreceptor that might affect activity. For example, if an animal cell line is used, species–specific differences in receptor or coreceptor expression levels and/or amino acid sequences may affect antibody activity. Likewise, if immortalized cell lines are used, results may vary among cell lines or fail to accurately reflect those obtained with primary cells. For example, several research groups reported that the neutralization activity of some anti-SARS-CoV-2 mAbs is affected by ACE2 expression levels, leading to variable potency (in terms of both the half-maximal effective concentration [EC_50_] values and maximal percentage inhibition) in different cell lines [153,159,160,161]. However, these studies are difficult to interpret because they, unfortunately, did not include relevant primary cell types as controls. If primary cells are used, it may be beneficial to test cells from multiple donors (ideally of different sexes and ethnicities) to assess variability in potency. For viruses that infect multiple cell types (e.g., herpesviruses), either due to expression of the same receptor on multiple cell types or use of multiple receptors, antiviral activity can be assessed in distinct cell types.

In addition to neutralization assays, cell type is an important factor to consider for other types of antibody assays, including assessments of Fc effector functions (e.g., ADCC, ADCP, CDC), ADE, and other types of studies. Characterization of the effector function is a consideration for both monoclonal and polyclonal antibodies. In general, these assays are not standardized, and it is often unclear which cell types and Fc effector functions are most likely to be relevant for clinical efficacy. To further complicate matters, Fc effector function assays often involve at least two cell types: target cells expressing the antigen and immune effector cells that respond to the IgG-bound antigen. In most cases, target cells consist of a cell line (e.g., CHO, Jurkat, or 293T) that has been transiently transfected or engineered to constitutively or inducibly express viral antigen(s), such as envelope protein. For example, Fc effector functions were assessed for the FDA-approved anti-EBOV mAbs (Inmazeb and Ebanga) using target cell lines with inducible expression of the EBOV glycoprotein [162,163]. Alternatively, virus or viral-like particles may be used as targets in some assays (e.g., for ADCP). For effector cells, common cell types used include Jurkat (immortalized human T) or NK-92 (immortalized human NK-like) cells engineered to stably express specific FcγRs and primary human cell types, such as NK cells, monocytes, monocyte-derived macrophages, or peripheral blood mononuclear cells. Many manufacturers developed reporter cell lines (often Jurkat-based) to quantify FcγR activation as a surrogate for ADCC, and some research was performed to compare these assays to classical ADCC assays [164,165]. Fc effector function assays can be performed with cells that express different FcγRs and FcγR variants, e.g., the FcγRIIIa F158 and V158 variants, which have distinct binding affinities for IgG1 and IgG3 [166].

The selection of cell lines for assessment of ADE is also an important issue, particularly for viruses in which ADE is known to be a significant issue (e.g., Dengue and Zika viruses). For these viruses, ADE was assessed using K562 (FcγRIIa+ human erythroleukemia), Raji (human B lymphoblastoid), U937 (human myeloid leukemia), THP-1 (human monocytic leukemia), and primary monocytes or macrophages [167,168,169,170]. In many cases, mAbs are engineered to have Fc substitutions that are expected to enhance, diminish, or abrogate Fc effector functions and/or ADE. In these cases, cell culture studies can be performed to verify that the substitutions have the intended effects. It may be beneficial to test multiple versions of a mAb in parallel to identify one with optimal properties, i.e., versions with an unmodified Fc region, different Fc substitutions, or distinct Fc glycosylation patterns. Lastly, cell lines for other types of studies should also be carefully selected, such as for studies of antibody resistance mechanisms, cell–cell transmission, and cell–cell fusion or syncytium formation.

### 3.3. Resistance

As part of development, it is critical to characterize the resistance mechanisms and pathways of antiviral antibodies in pre-clinical (and later clinical) studies. Antiviral resistance can develop through a variety of mechanisms [171,172]. Naturally occurring resistance results from polymorphisms that arise as the virus naturally evolves, as strains with increased fitness (e.g., due to enhanced infectivity, replication, or immune escape) become dominant in the human population. This type of resistance is unrelated to treatment but may lead to treatment failure or non-response. In contrast, treatment-emergent resistance happens in response to the specific antibody treatment, with treatment providing the selective pressure for the emergence of the resistant variant. The potential for antiviral antibody therapies to be affected by either form of resistance should be assessed in pre-clinical studies [173]. These studies are often highly valuable for informing the dose and dosing interval, optimization of treatment regimens (e.g., mAb monotherapy vs. mAb combinations vs. mAbs+other antivirals), interpretation of clinical resistance data, identification of patients infected with susceptible viral variants, determination of likelihood of cross-resistance with other mAbs (important for rescue/salvage therapy), and conducting genomic surveillance efforts. Approaches for resistance characterization vary widely depending on the virus, antibody, and antibody target. For example, approaches for SARS-CoV-2 include testing the effects of single amino acid substitutions in the spike protein (S) on antibody binding in biochemical assays, screening large libraries of S proteins with substitutions using a yeast display system, determining the effects of S substitutions on antibody neutralization in cell culture using pseudotyped VLPs, and performing resistance selection in cell culture and animal models with replication-competent chimeric (i.e., VSV-spike) and authentic viruses [155,174,175,176,177]. For mAbs that target host proteins, resistance could potentially arise from genetic polymorphisms that alter the expression or sequence of the host protein, although this was considered unlikely for ibalizumab [178]. In these cases, differences in host genetics (e.g., single-nucleotide polymorphisms) can be assessed through bioinformatics and, if necessary, biochemical or cell culture studies.

Some studies performed to assess resistance, particularly those involving the selection or characterization of replication-competent authentic or recombinant viruses, can raise significant ethics and biosecurity concerns. These studies are conducted only under appropriate biocontainment and in strict accordance with all applicable institutional, local, regional, and national guidelines and regulations. In some cases, it may be possible to adequately characterize antibody resistance using biochemical or cell culture assays that involve only the viral antigen or a replication-defective virus. In other cases, it may be possible to select for antibody resistance using a replication-competent virus that poses less risk, such as a replication-competent chimeric virus, a related animal virus that expresses the same epitope but cannot infect human cells, or a deliberately attenuated version of a human virus. One caveat of these approaches is that the substitutions observed in these viruses may not accurately predict the resistance substitutions observed with authentic viruses. When an authentic virus must be used due to unavailability of other approaches or inadequate resistance information from such approaches, risk can be mitigated using an authentic virus that is susceptible to current vaccines and antivirals and to which vaccine-induced or natural immunity is already widespread.

### 3.4. Animal Studies

Animal studies can be a powerful tool for assessing the safety and efficacy of antiviral antibody therapies prior to commencing clinical trials. To be useful, an animal model should recapitulate as closely as possible the critical aspects of human disease, including susceptibility to the pathogen, route of infection, viral tropism, severity of outcomes, pathophysiology of systemic and end organ disease as applicable, and host responses. Although non-human primates, being evolutionarily closest to humans, are most likely to fulfill these criteria, many other mammalian species were successfully used as models of viral disease, including for assessing antibody therapies. Examples include mice for West Nile virus, ferrets for influenza, cotton rats for RSV, and hamsters for hantavirus and SARS-CoV-2 [179].

The role of animal studies in the development of antiviral therapies can be illustrated by the recent COVID-19 pandemic. For SARS-CoV-2, initial cell culture studies focused on identifying pathways underlying viral entry, tropism, molecular pathways of disease processes, and mechanisms for neutralization in cell lines and organ-like systems, with the ultimate goal being the discovery of effective preventive and therapeutic strategies, including antibody therapies. Although critical for identifying and measuring neutralization activity of antiviral antibodies, cell culture studies such as these cannot account for in vivo activity or their distribution in the mucosal or lung tissue—often the point of entry and viral replication for SARS-CoV-2. Studies in animal models, including hamsters, transgenic mice, ferrets, and non-human primates, validated the findings from cell culture studies and demonstrated the potential for efficacy, including of anti-SARS-CoV-2 antibodies [180,181,182,183,184,185,186,187]. Similar studies were used to support EUA packages for mAbs that received such authorizations [72]. These studies demonstrated that monoclonal and polyclonal antibodies have the potential to protect against disease when used as prophylaxis and improve outcomes when used as a therapeutic, thus providing preliminary data to support their investigation in clinical trials. They also demonstrated that ADE was not observed.

A critical question for advancing any therapeutic modality in clinical trials is related to the starting dose, which should be both safe and potentially efficacious. Although the definite proof of a safe and effective dose will come from well-designed and adequately controlled clinical trials, a scientific and data driven rationale should inform the dose(s) chosen to advance to such trials. Data supporting safety are obtained from non-clinical toxicology, safety pharmacology, and other pertinent studies, as outlined in the appropriate FDA guidance documents [188]. Deriving a potentially efficacious dose, on the other hand, is not as standardized, and multiple approaches can be applied based on the availability of cell culture and animal models, new or existing pharmacokinetic (PK) and pharmacodynamic (PD) data in both animals and humans, physiologically based pharmacokinetic (PBPK) models, and biomarkers that correlate with efficacy. To continue with the example of the recent pandemic, the data submitted by sponsors in EUA packages shed light on some of the methods used [72]. For all these antibodies, a necessary component, and often the first step in efficacious dose estimating, was identification of the antibody concentration providing 90% of maximal effect (EC_90_ value), followed by target clinical exposures that exceed this level, taking into account antibody distribution in the respiratory tract. Animal data can assist in deriving such concentrations, either through direct measurements or methods that relate serum to tissue concentrations, such as antibody biodistribution coefficient or allometric scaling [189]. It should be noted that anatomical and physiological characteristics differ depending on the species and the degree of phylogenetic similarity with humans. Thus, biodistribution of the antibody to the site of action (such as lung or gastro-intestinal tract) may be quite different. This difference may also be further influenced by any variability in disease presentation and pathological processes.

Other considerations that influence the translatability of efficacy data from animal studies include differences in expression patterns of Fc receptors on effector cells and the affinity of the human antibodies to the animal orthologs [190,191]. In addition, Fc modifications intended to alter FcRn- or FcγR-binding may not have the same effects in animals and humans. For example, M252Y/S254T/T256E, referred to as “YTE” variant, has a half-life four times longer than unmodified IgG1 in humans but a rapid clearance in rodents [72,192]. To overcome these limitations, transgenic and humanized mouse models that incorporate human FcγR genes were developed [193,194].

In certain infectious disease settings, specifically when clinical trials are not feasible or ethical, adequate and well-controlled animal studies can be used to provide substantial evidence for efficacy through a pathway known as approval under the Animal Rule. Detailed advice on considerations when developing a therapy under this pathway are described in the pertinent FDA guidance [195]. The guidance outlines many clinical and non-clinical aspects of such programs, including considerations for choosing the animal models, such as the challenge agent, susceptibility to disease, mechanisms of virulence, pathophysiology and its comparison with human disease (natural history studies), triggers for intervention, mechanisms of action for the treatment, the dose, and the necessary studies that can be used to derive a dose and dosing regimen in humans (such as PK and/or PD studies in animals and humans). It should be noted that seeking regulatory approval under the Animal Rule is not a way to circumvent performing clinical studies or simplify the approval process for a therapeutic. Safety and PK studies in humans are still needed, whereas performing studies to adequately characterize at least two animal models, often undertaken under high containment conditions in BSL-4 facilities, adds significant complexity to this process. This fact is reflected in the number of approvals: a total of 16 drugs were previously approved under this rule but none of them are antiviral antibodies, although there are four antibodies targeting bacterial infections or toxins [196].

## 4. Combining Antiviral Antibodies and Other Therapies

In the following sections we will discuss combination therapies containing antiviral antibodies, highlighting potential benefits and challenges. We will also provide examples from clinical practice and the scientific literature regarding the use of antiviral antibodies with other drugs or biologics. The intent is not to endorse any specific combinations, but to give a panoramic view of the potential for treatments that, if proven safe and effective, can help combat viral diseases and make an impact on public health.

### 4.1. Combinations of Specific Polyclonal Antibodies with Vaccines or Drugs

The combination of SpIG with vaccines for pre- or post-exposure prophylaxis is well-established, dating back to historical use of live vaccinia virus for smallpox vaccination, in combination with Vaccinia Immune Globulin, to prevent complications in patients with risk factors for serious adverse events, such as eczema vaccinatum [197]. In contrast, vaccine-SpIG combinations for rabies [198], HAV [199], and HBV [54,55,56] are used to prevent viral spread until host vaccine responses are fully developed. In these settings, SpIG is effective if given early enough after exposure, when viral burdens are low and before effective vaccine responses can develop. This “window of opportunity” to neutralize virus with SpIG so as to prevent viral spread is defined for passive immune therapies for rabies (0–7 days) [44,45,46], HAV (0–2 weeks) [53], measles (0–6 days) [53], HBV (0–14 days; 0–12 h for infant born to infected mother; as soon as possible for recipient of HBV+ blood) [55,56], and varicella zoster (0–4 days) [47].

Several principles are common to vaccine-SpIG combination treatments. Firstly, the vaccine and SpIG, if given at the same time, should always be administered at different anatomic sites (separate limbs). Secondly, live vaccines should be withheld for 3–6 months after SpIG because other antibodies contained in the IG products may interfere with effectiveness.

The combination of SpIG and drugs is most often used in transplant settings, where the goal is suppression of viral activation and clinical disease and prevention of organ damage. The combination of SpIG and an antiviral drug provides orthogonal methods for viral control or clearance. For example, CMVIGIV is often used in combination with ganciclovir or similar drugs to prevent cytomegalovirus disease associated with transplantation of kidney, lung, liver, pancreas, or heart [41]. Acyclovir and related drugs are reportedly used on occasion with varicella IG, based on clinical judgement, though there are no previous clinical trials directly comparing VARIZIG alone or with the addition of acyclovir [49]. Hepatitis B virus (HBV) Immune Globulin (Hepagam B only) is licensed as a monotherapy to prevent recurrence of HBV in HBV-infected liver transplant recipients [56]. However, clinical guidelines recommend use of HBIG + nucleoside analogs for liver transplant patients at higher risk for endogenous HBV recurrence, or to prevent transmission of HBV from a transplanted organ (infected donor) [57]. Several nucleoside analogs are now licensed for treatment of HBV. Finally, Vaccinia Immune Globulin (VIG) was used to treat complications stemming from smallpox vaccination. In immunocompromised SCID mouse models of progressive vaccinia, frequent administration of VIG staves off clinical signs of disease and death. However, when treatment stops, the mice succumb to viral infection after several months [200]. Progressive vaccinia that occurs in immunocompromised patients after vaccinia exposure may be slowed or halted through VIG, although complete resolution of infection is thought to coincide with improved function of the immune system based on anecdotal reports [201]. The ability of VIG combined with cidofovir to eliminate vaccinia in a proportion of SCID mice suggests that antibody plus drug treatment could be advantageous for severely immunocompromised patients [200]. Generally, treatments for eczema vaccinatum and progressive vaccinia are combined with investigational antivirals and/or cidofovir [50,51]. Controlled human studies of adjunct drug treatments are not currently feasible due to the rarity of severe vaccinia infections.

### 4.2. Combinations of Monoclonal Antibodies

The first mAb combination approved for a viral disease, known as Inmazeb, consists of three mAbs that have non-overlapping epitopes and can simultaneously bind to EBOV glycoprotein: atoltivimab, maftivimab, and odesivimab [163]. All three mAbs are human IgG1 antibodies without substitutions in the Fc regions. In non-clinical assays, these mAbs have distinct mechanisms of action: atoltivimab neutralized virus and mediated Fc effector functions, including FcγRIIIa activation (used as a surrogate of ADCC) and ADCP. Maftivimab neutralized virus but did not activate FcγRIIIa or ADCP. Lastly, odesivimab did not neutralize virus but activated FcγRIIIa and ADCP [163]. In addition, as noted above, several mAb combinations were previously authorized by the FDA for the pre-exposure prophylaxis, post-exposure prophylaxis, or treatment of COVID-19: casirivimab+imdevimab (REGEN-COV), bamlanivimab+etesevimab, and cilgavimab+tixagevimab (Evusheld). Casirivimab and imdevimab are human IgG1 antibodies with unmodified Fc regions that target non-overlapping spike epitopes, neutralize virus, and activate ADCC and ADCP [202]. Bamlanivimab and etesevimab are human IgG1 antibodies with unmodified (bamlanivimab) or LALA-modified (etesevimab) Fc regions that target partially overlapping spike epitopes, neutralize virus, and activate FcγRIIIa (bamlanivimab only) [203]. Cilgavimab and tixagevimab are human IgG1 antibodies with YTE-TM-modified Fc regions that extend antibody half-life, reduce Fc effector functions, and minimize the risk of ADE [204]. They target non-overlapping spike epitopes, neutralize virus, and do not mediate Fc effector functions in cell culture. In addition, mAb combinations are currently under development for many other viruses, including CMV, HIV-1, influenza virus, and rabies virus [63,205,206,207,208]. For general regulatory advice on the codevelopment of mAbs, we encourage readers to refer to the FDA guidance: “Codevelopment of Two or More New Investigational Drugs for Use in Combination”, which applies only to drugs and biologics regulated by CDER [209].

#### 4.2.1. Potential Benefits of Monoclonal Antibody Combinations

Synergy. The antiviral activity of antibody combinations in cell culture may be additive [210], antagonistic [211] (as discussed below), or synergistic [212]. For example, several mAb combinations with synergistic neutralization activity have been identified for HIV-1 and SARS-CoV-1 [213,214,215,216]. Relative to individual mAbs, synergistic interactions between mAbs could lead to enhanced antiviral activity or comparable activity at lower concentrations, potentially leading to reduced or less frequent dosing and fewer adverse reactions in patients. In some cases, mAb combinations might also have synergistic effects on viral replication or disease in patients. However, in clinical trials of mAb combinations for viral diseases, individual mAbs are often not tested due to concerns about resistance, making it impossible to determine whether the combinations are synergistic in humans. Thus, these types of studies are often best performed in animal models.

Greater Breadth of Activity. As individual mAbs are highly specific for a single epitope, their activity can be significantly affected via naturally occurring amino acid polymorphisms in or near the epitope. Thus, mAb combinations are often designed to target non-overlapping epitopes on the same viral protein, leading to broader activity against different virus types, genotypes, subtypes, and/or variants than individual mAbs. For example, the anti-rabies virus mAbs R172 and R173, which target non-overlapping sites on the viral glycoprotein, are being developed in combination to ensure sufficient breadth of activity against viral variants circulating in North America [205]. However, mAb combinations were also developed to target different viral proteins or overlapping epitopes on the same viral protein. For example, the anti-CMV mAbs LJP538 and LJP539 target different viral proteins, while the anti-SARS-CoV-2 mAbs bamlanivimab and etesevimab target partially overlapping epitopes on the same protein [203,217]. In addition to the number of mAbs, epitope conservation is an important factor to consider. In principle, a single mAb that targets a highly conserved epitope could have broader activity than a combination of several mAbs that target poorly conserved epitopes.

Reduced Likelihood of Resistance. Relative to individual mAbs, combinations of mAbs are generally less susceptible to the development of treatment-emergent resistance, as resistance would likely require amino acid changes in or near both epitopes. Thus, resistance may develop less frequently or with delayed kinetics relative to individual mAbs. For example, in several studies of anti-SARS-CoV-2 mAbs, resistance was readily selected in cell culture with single mAbs but not combinations of mAbs targeting non-overlapping spike epitopes [174,175,177]. In a clinical trial, SARS-CoV-2 resistance emerged less frequently with a mAb combination (bamlanivimab+etesevimab) than with one of the mAbs alone (bamlanivimab), although it should be noted that these mAbs target partially overlapping epitopes, and an etesevimab-only control arm was not included [218]. Likewise, HIV-1 resistance to mAbs is thought to be less likely to develop clinically with mAb combinations as opposed to single mAbs [219]. We note that this potential benefit of mAb combinations may only apply when both mAbs are active against the viral variant present at the time of treatment initiation.

Multiple Mechanisms of Action. Like individual antibodies, mAbs in combinations may have multiple mechanisms of action, including neutralization and Fc effector functions. However, in contrast to individual antibodies, mAbs in combinations can be designed to specialize in different functions. This is perhaps best illustrated by Inmazeb, which, as described above, contains three mAbs: one with only neutralization activity, one with only Fc effector function activity, and one with both activities in cell culture [163]. However, the relative contribution of each antibody and mechanism of action to clinical efficacy is often unclear (see below).

#### 4.2.2. Potential Challenges of Monoclonal Antibody Combinations

Resistance. In principle, mAb combinations are expected to have broader activity and be less susceptible to resistance than individual mAbs. However, these issues also represent significant challenges for mAb combinations. For example, most anti-SARS-CoV-2 mAb combinations were found to have significantly reduced activity in cell culture against the SARS-CoV-2 Omicron BA.1 variant, which emerged in November 2021. According to a recent review, casirivimab+imdevimab (REGEN-COV), bamlanivimab+etesevimab, and cilgavimab+tixagevimab (Evusheld) had 840-, 740-, or 75-fold reduced neutralization activity (based on geometric mean EC_50_ values), respectively, against BA.1 in cell culture [220]. As another example, in a recent Phase 1 clinical trial of a combination of three anti-gp120 mAbs for the treatment of HIV-1, participants had transient decreases in viral load, followed by a rebound in viruses with partial or complete resistance to two of the mAbs in cell culture [221]. The authors hypothesized that at least four broadly neutralizing mAbs may need to be combined for HIV-1 treatment to provide broad activity and prevent resistance.

Uncertain Contribution of Each mAb to Clinical Efficacy. In mAb combinations for viral diseases, it is often unclear to what extent each mAb contributes to clinical efficacy. The FDA generally requests sponsors who are developing combinations of two or more investigational drugs to demonstrate that the combination is more effective than each single drug acting alone [209]. However, non-clinical data demonstrating that the combination is superior to the single drugs in some aspect (e.g., better activity or less resistance) can be considered sufficient when the combination is intended to treat a serious disease or condition and there is a strong rationale for the use of the combination (e.g., prevention of resistance). In the cases of FDA-approved and previously authorized mAb combinations for viral diseases, individual mAbs were generally not tested in the trials that evaluated clinical efficacy. Thus, there is significant uncertainty about the extent to which each mAb contributes to clinical efficacy, as well as whether the mAb combination would retain efficacy against viral variants resistant to one of the mAbs in cell culture.

Uncertain Contribution of Neutralization vs. Fc Effector Functions to Clinical Efficacy. For individual antibodies, mAbs in combinations often have multiple mechanisms of action that include neutralization and Fc effector functions, unless the Fc regions have been modified to disrupt effector functions. Thus, it is often unclear to what extent neutralization and Fc effector functions contribute to clinical efficacy. Likewise, it is often unclear if a mAb combination found to lack neutralization activity against a particular viral variant in cell culture could at least partially retain clinical efficacy through Fc effector functions. To further complicate matters, cell culture assays to assess neutralization and Fc effector functions are poorly standardized, and it usually unclear which Fc effector functions or assays are the most relevant to clinical efficacy. Such challenges have frequently arisen with anti-SARS-CoV-2 mAbs due to the continued emergence of novel variants with varying degrees of susceptibility to neutralization using mAbs and mAb combinations. These issues can be addressed in animal models through comparing different versions of mAbs (e.g., unmodified Fc vs. modified Fc with disrupted or enhanced FcγR binding), which was carried out for SARS-CoV-2 [185,222,223,224]. However, the extent to which animal models can predict clinical efficacy remains unclear, as animals have differences in FcγRs and IgG-FcγR interactions compared to humans.

Other Challenges. In addition to the issues described above, other challenges for the development of mAb combinations for viral diseases include the potential for antagonism between mAbs [220], particularly those with partially overlapping epitopes; low distribution of mAbs to some sites of interest (e.g., 6.5–15% for lung epithelial lining fluid) [225,226,227]; lack of standardization of non-clinical assays and reagents, leading to highly variable results across assays [220]; potential diminishment of mAb activity by soluble antigen (or subviral or virus-like particles) [228,229]; potential for cross-resistance with other mAbs that have the same target; potential interference with diagnostic assays [230]; potential attenuation of the immune response after vaccination or infection; potential for the development of anti-drug antibodies; and uncertainty about the optimal dose and, in the case of repeated administration, dosing schedule.

In addition to challenges related to clinical outcomes, there are some chemistry, manufacturing, and control (CMC) challenges related to co-formulated mAbs. These challenges include methods that can identify all mAbs in the drug product and that they are present at a consistent ratio in each lot; and understanding the nature of aggregates due to co-formulation and high concentrations. Multiple potency assays may be needed if the mAbs have different mechanisms of action, for example neutralization or Fc-mediated effector functions. Another challenge from both the CMC and clinical standpoints arises when drug product is diluted in an IV solution and large volumes are infused. The contribution of potential endotoxin from both the drug product and diluent should be considered. The compendial limit for infusion solutions is not more than 0.5 endotoxin units (EU) per mL [231]. Depending on the volume of the diluted drug product to be delivered, the endotoxin release criteria for the drug product may need to be adjusted to comply with the endotoxin limits for patients of less than 5.0 EU/kg/hour.

### 4.3. Combinations of Monoclonal Antibodies with Other Types of Antivirals

In addition to mAb combinations, there are many examples of mAbs and mAb cocktails being used in combination with other types of antivirals for viral diseases (Table 4). These mAbs target either a viral glycoprotein or a host receptor or co-receptor. The antivirals include approved drugs (e.g., oseltamivir), approved drugs being studied against a different virus (e.g., remdesivir), and investigational drugs (e.g., VIR-2218, a small-interfering RNA [siRNA]). These antivirals belong to many different classes, including nucleos(t)ide analog reverse transcriptase inhibitors, RNA-dependent RNA polymerase inhibitors, protease inhibitors, capsid inhibitors, neuraminidase inhibitors, fusion inhibitors, coreceptor antagonists, therapeutic vaccines, latency-reversing agents, and immunomodulators. In most cases, the mAbs and antivirals target either different viral proteins or a viral protein and a host protein.

As is the case for mAb cocktails, the major benefits of combinations of mAbs and other antiviral drugs include the potential for synergy, broader activity against different viral variants, and reduced likelihood of resistance. Many of the combinations listed in Table 4 were reported to have synergistic effects on viral replication or disease progression in cell culture and/or animal models. For example, several mAbs targeting HCV receptors were found to exhibit synergistic activity against HCV when combined with approved drugs for HCV in cell culture and human liver-chimeric mice [234]. Likewise, several mAbs targeting HA were found to exhibit synergistic activity against IAV when combined with oseltamivir, which is an FDA-approved influenza neuraminidase inhibitor, in mice and ferrets [241,242,243,244]. While little clinical data are available for most of these combinations, the combination of VIR-3434, which is a mAb that targets HBsAg, and VIR-2218, which is an siRNA that targets HBV gene expression, was found to have more enhanced antiviral activity (in terms of serum HBsAg reductions) than either drug alone in a phase 2 trial [232]. In other studies, combinations of mAbs targeting the MARV or SUDV glycoproteins and remdesivir were found to extend the therapeutic window of antiviral therapy in MARV- or SUDV-infected rhesus macaques [239,240]. The authors hypothesized that such combinations might also extend the therapeutic window in humans. Other potential benefits of these combinations include enhanced antiviral activity across different tissues due to different drug distribution profiles, broader antiviral activity, shorter treatment schedules, reduced doses, and lower risk of adverse events due to reduced doses or dosing durations. For some viral diseases, these combinations may not have major benefits relative to the individual drugs for the general patient population; however, they could still prove useful for specific sub-populations, such as patients who are immunocompromised or transplant recipients; cannot tolerate, do not respond, or have contraindications to standard-of-care therapies; or have drug-resistant virus or severe disease.

Challenges regarding the development of combinations of mAbs and other antiviral drugs can include insufficient breadth of antiviral activity, the development of resistance, and the uncertain contribution of each drug to clinical efficacy, particularly in cases where the individual drugs cannot be tested alone (e.g., due to concerns about resistance). For example, although mAbs and other antiviral drugs usually target different proteins, resistance may still arise because the exposure of one drug in a particular tissue is low or the patient is infected with a viral variant that is already resistant to one of the drugs at the time of treatment initiation. In other cases, these combinations may fail to improve antiviral activity or clinical efficacy relative to the individual drugs but lead to higher rates of adverse events. Combinations that include mAbs or antiviral drugs that target host proteins may result in toxicities or have variable activity across patients due to differences in host genetics. Given that mAbs and other antiviral drugs will usually have different dosage forms and half-lives, it may also be difficult to determine the optimal dosing regimen or the regimen might be complex, leading to problems with patient adherence and increasing the chance of drug resistance.

## 5. Future Directions and Conclusions

As the applications of antiviral antibody therapies expand, there are several areas that can benefit from further development. The international standardization of assays and reagents (e.g., viruses and cell lines) for measuring antibody activity could help address the variability in potency often observed for the same antibody in different assays or laboratories (e.g., 10–100-fold range in EC_50_ values for anti-SARS-CoV-2 mAbs) [220]. In addition, more work is needed to better understand the role of Fc effector functions in viral diseases using cell culture, animal models, and clinical studies. For example, it is not known if less understood mechanisms, such as antibody-dependent cellular trogocytosis, which has been demonstrated predominantly for mAbs developed for oncology indications [245], may also be contributors to antiviral activity. Trogocytosis is shown for some anti-HIV [246,247,248] and anti-SARS-CoV-2 [249] mAbs, but additional studies are needed to understand its contribution to overall activity of these mAbs and how broad a mechanism it may be across viral diseases. Furthermore, pre-clinical assays are being developed that are more physiologically relevant, especially potency assays that capture multiple functions of the antibody. For example, organ-on-a-chip and microphysiological systems can incorporate multiple cell types, including immune cells; simulate blood flow and organ perfusion; and provide data that serves as a bridge between standard cell culture assays and clinical studies [250]. Although still early in development and not commonly used in regulatory applications, such technologies are expected to become increasingly powerful and more widely used. These systems can also help address ethical concerns and societal pressures to replace, reduce, and refine animal research, and they have the potential to provide information that is more predictive of clinical efficacy.

Another area with unharnessed potential, especially for SpIG therapies, is the selection of specific glycosylation signatures to modulate downstream immune responses [251]. These strategies were proposed for use in the setting of autoimmune disease, but they could also potentially be applied to viral diseases. When combined with other novel technologies, such as the production of recombinant IG preparations [252], such methods have the potential to result in antiviral antibody preparations with improved properties.

Production of SpIG from convalescent plasma in a pandemic setting remains time-consuming and challenging. Convalescent plasma is often the earliest available antibody-containing treatment that could be effective for prevention of severe disease. Advances in technologies that can be used to rapidly and inexpensively select donations containing high titers of neutralizing antibodies from among thousands of donations are needed both for direct use of convalescent plasma and manufacturing of SpIG. Biosensor-based methods that reliably measure neutralizing potency in plasma donations and products, and that can be used in a low biocontainment (BSL-2) setting, are promising. In addition to improved donor screening, technical advances in manufacturing that would maximize the yield of SpIG during a pandemic could include affinity matrices or changes in manufacturing steps to allow virus-specific IgM to copurify with IgG.

For mAbs, large volumes of product are usually infused intravenously over several hours. The length of infusion time may depend on the amount of mAb needed per body weight and whether a patient is experiencing infusion-related reactions typical of mAbs. One strategy that was previously used to address administration barriers was co-formulating a high concentration of the mAb with recombinant human hyaluronidase [253]. This enzyme degrades hyaluronic acid in the extracellular matrix, facilitating rapid delivery of large volume subcutaneous injections and increasing the bioavailability of the product. This outcome was previously accomplished with several mAbs for oncology, including the combination of rituximab, trastuzumab, daratumumab or trastuzumab, and pertuzumab with recombinant human hyaluronidase [254]. This approach is being studied with an anti-HIV-1 mAb (clinicaltrials.gov #NCT03538626 and [208,255]). These formulations provide more convenient dosing for patients, but must be supported with adequate non-clinical and clinical safety data.

Other developments for anti-viral mAbs include bispecific antibodies [256], single domain antibodies derived from camelids [257], and other scaffold proteins, such as DARPIns [258] and Adnectins [259], which are engineered in the loop regions between more structured regions of the core domain to mimic antibody CDRs. Some of these technologies may be able to target epitopes that are difficult for traditional antibodies to recognize. Furthermore, these novel constructs may be more cost effective to manufacture than mAb cocktails, and lower doses may be as effective as higher doses of a mAb cocktail. However, clinical studies are needed to determine efficacy and safety and to see if there are issues, such as immunogenicity, related to these novel products.

Whether alone or in combination, antiviral antibody therapies can provide important prophylaxis and treatment options to help relieve the burden of viral diseases. This space is rapidly evolving, and, as more experience is gained through successful clinical applications, the products of the future have the potential to overcome many of the challenges we describe, while continuing to fulfill the promise of safety and effectiveness.

## Figures and Tables

**Table 1:** FDA-approved human polyclonal antibodies for prevention or treatment of viral diseases.

Target	Trade Name(s)	Donors	Indications	Used With
Rabies [44,45,46]	HyperRAB, Imogam, KedRab	Vaccinated	Post-exposure prophylaxis	Rabies vaccine (required, see prescribing information for rabies IG)
Varicella [47]	VARIZIG	Donations selected from high-titer donors after natural infection *	Post-exposure prophylaxis in patients at risk for severe infection	Concomitant use of acyclovir reported to occur in clinical practice [48]
Vaccinia [49]	Vaccinia Immune Globulin (Human)	Vaccinated	Treatment of severe complications after smallpox vaccination	Investigational antiviral drugs and/or cidofovir [50,51]
Cytomegalovirus (CMV) [41]	CytoGam	Donations selected from source plasma	Prevention of CMV disease in patients receiving organ transplants from CMV donors	Ganciclovir recommended in prescribing information; other drugs recommended in practice guidelines [52]
Hepatitis A (HAV) [53]	GamaSTAN	Regular donors	Pre- and post-exposure prophylaxis	None
Hepatitis B (HBVIG/IGIV) [54,55,56]	HyperHEP B, Nabi-HB	Vaccinated	Post-exposure prophylaxis	None
	HepaGam-B	Vaccinated	Post-exposure prophylaxis Prevention of HBV recurrence in HBsAg+ liver transplant recipients	Concomitant treatment with other drugs recommended in practice guidelines [57]
Measles [53] ^†^	GamaSTAN	Regular donors	Prevention or attenuation of measles in susceptible individuals	None
Rubella [28]	GamaSTAN	Regular donors	To modify rubella in exposed pregnant women who will not be undergoing a therapeutic abortion	None

* GamaSTAN may be used only if VariZIG is not available [53]. ^†^ In patients receiving IG products to correct antibody deficiencies, doses of intravenous IG (IVIG) that should prevent measles infections for travelers to measles-endemic areas are suggested [58].

**Table 2:** FDA-approved monoclonal antibodies for prevention or treatment of viral diseases.

Non-Proprietary Name	Trade Name	Target	Indication
palivizumab	Synagis	RSV F protein	For the prevention of serious lower respiratory tract disease resulting from RSV in pediatric patients (specific conditions and age limitations)
ibalizumab	Trogarzo	CD4 (post-attachment HIV-1 inhibitor)	In combination with other antiretroviral(s), for the treatment of HIV-1 infection in heavily treatment-experienced adults with multidrug-resistant HIV-1 infection failing their current antiretroviral regimen
atoltivimab, maftivimab, odesivimab-ebgn	Inmazeb	Ebola virus glycoprotein	For the treatment of infection resulting from *Zaire ebolavirus* in adult and pediatric patients, including neonates born to a mother who is RT-PCR positive for *Zaire ebolavirus* infection
ansuvimab-zykl	Ebanga	Ebola virus glycoprotein	For the treatment of infection resulting from *Zaire ebolavirus* in adult and pediatric patients, including neonates born to a mother who is RT-PCR positive for *Zaire ebolavirus* infection

**Table 3:** Advantages and disadvantages of specific polyclonal and monoclonal antibodies for prophylaxis or treatment of viral diseases.

	Specific Polyclonal Antibodies	Monoclonal Antibodies
Advantages	React with multiple target epitopes on the same or different viral proteins Contain more than one type of neutralizing antibody Less susceptible to resistance Potential for higher avidity Mix of IgG subclasses provides expanded potential for Fc effector functions	Highly specific for a single well-defined epitope Can be engineered in V domain for affinity, avidity, specificity, and/or the Fc region to enhance or abrogate Fc effector functions or extend half-life. Can combine multiple mAbs targeting different epitopes or proteins Can be manufactured and released using platform strategies, which allow rapid entry into clinical trials Batch-to-batch consistency
Disadvantages	Large potentially variable pool of donors who must be screened for pathogen safety Potential for antibody dependent enhancement of infection/disease May dampen immune responses after vaccination with live viruses (e.g., measles, mumps, etc.). Can interfere with diagnostic assays Presence of low amounts of other plasma proteins, which could contribute to adverse events	Individual mAbs susceptible to resistance Potential for antibody dependent enhancement of infection/disease May dampen immune responses after vaccination Can interfere with diagnostic assays Can induce the formation of anti-drug antibodies

**Table 4:** Examples of combinations of mAbs and other types of antivirals.

Virus	mAb(s) (Target)	Antiviral(s)	Stage	References
HBV/HDV	VIR-3434 (HBsAg)	VIR-2218 ± NrtI ± pegIFN	Phase 2	NCT04856085 *, also see [232,233]
HCV	various (HCV receptors) ^†^	Various ^†^	Pre-clinical	[234]
HIV-1	Teropavimab + zinlirvimab (gp120)	Various ^‡^	Phase 1–2	NCT04811040 *, also see [235,236,237]
HIV-1	Ibalizumab (CD4)	Approved antiretrovirals	Approved	[178]
IAV	Various (HA) ^§^	Oseltamivir	Phase 2	[206]
IAV	CR9114 + F3A19 (HA)	Favipiravir	Pre-clinical	[238]
MARV	MR186-YTE (GP)	Remdesivir	Pre-clinical	[239]
SUDV	ADI-15878 + ADI-23774 (GP)	Remdesivir	Pre-clinical	[240]

* Clinicaltrials.gov study number ^†^ mAbs tested were OM-7D3-B3 (anti-CLDN1 mAb), NK-8H5-E3 (anti-SR-BI mAb), and QV-6A8-F2C4 (anti-CD81 mAb). Antivirals tested were HCV NS3/4A protease inhibitors (simeprevir, danoprevir, boceprevir, telaprevir), NS5A inhibitors (daclatasvir), and NS5B nucleotide analog polymerase inhibitors (sofosbuvir). ^‡^ Therapeutics being tested in combination with these mAbs in clinical trials include FDA-approved antiretrovirals (e.g., the HIV-1 capsid inhibitor lenacapavir) and investigational drugs, such as peptide fusion inhibitors, therapeutic vaccines, latency-reversing agents, and immunomodulators (e.g., pegylated interferon). ^§^ mAbs tested in combination with oseltamivir in clinical trials include CT-P27 (a combination of two mAbs), MEDI8852, MHAA4549A, and VIS410. Abbreviations: GP, glycoprotein; HA, hemagglutinin; HBsAg, hepatitis B virus surface antigen; HBV, hepatitis B virus; HCV, hepatitis C virus; HDV, hepatitis delta virus; HIV-1, human immunodeficiency virus type-1; IAV, influenza A virus; mAb, monoclonal antibody; MARV, Marburg virus; NrtI, nucleos(t)ide analog reverse transcriptase inhibitor; pegIFN, pegylated interferon-α; and SUDV, Sudan virus.

## Data Availability

Data sharing not applicable.
